# Molecular Aspects in the Potential of Vitamins and Supplements for Treating Diabetic Neuropathy

**DOI:** 10.1007/s11892-021-01397-1

**Published:** 2021-08-27

**Authors:** Tina Okdahl, Christina Brock

**Affiliations:** 1 Mech-Sense, Department of Gastroenterology and Hepatology, Aalborg University Hospital and Clinical Institute, Aalborg University, Mølleparkvej 4, 9000 Aalborg, Denmark; 2 Department of Clinical Medicine, Aalborg University, Aalborg, Denmark

**Keywords:** Diabetic neuropathy, Dietary supplement, Antioxidants, Neuroprotection

## Abstract

**Purpose of Review:**

To discuss and provide evidence-based data on dietary supplements as part of treating diabetic neuropathy

**Recent Findings:**

Few randomized controlled trials are available, but some have shown beneficial efficacy of various dietary supplements on objective primary endpoints including nerve conduction velocities and axon potentials as well as subjective patient-reported outcomes.

**Summary:**

No medical cure for diabetic neuropathy exists, and prevention is therefore crucial. Tight glucose control slows the progression of nerve damage in diabetes, but an unmet clinical need for effective interventions is warranted. Consequently, a growing number of patients turn to dietary supplements proposed to possess neuroprotective properties. However, the postulated effects are often not evidence-based as they have not been tested scientifically. Taken together, this review will focus on dietary supplements investigated in clinical trials for their potential capabilities in targeting the molecular mechanisms involved in the underlying pathogenesis of diabetic neuropathy.

## Introduction

The incidence of people diagnosed with diabetes worldwide is escalating. Meanwhile, treatment has improved, thus leading to longer life expectancies for affected individuals. Accordingly, the presence of long-term diabetes and macro- and microvascular complications is increasing [1]. Currently, it is considered as one of the most important public health issues, as these complications cause negative impact on the individual quality of life and increase socioeconomic expenditure. Among the microvascular complications, polyneuropathy affects up to 50% of adults with long-term type 1 and type 2 diabetes. The pathogenesis is complex and multifactorial and includes immune-mediated, inflammatory, vascular, and metabolic pathways [2, 3]. The clinical manifestations of classical diabetic symmetrical polyneuropathy (DSPN) include sensory and motor loss of the peripheral nerves to feet and hands, evident as a “stocking-and-glove” distribution mirroring a length-dependent axonopathy of the larger sensory and motor nerves in the extremities [4]. Thus, patients experience a number of paradoxical sensory deficits ranging from loss of protective sensation/numbness to debilitating neuropathic pain [5].

## Proposed Molecular Pathways Involved in Diabetic Neuropathy

Nerves are particularly sensitive to fluctuations in blood glucose levels because neurons have continuously high glucose demand and are vulnerable to episodes of glycolytic and anaerobic metabolism. Furthermore, neurons fail to regulate episodic glucose uptake under the influence of insulin. Neurons express the glucose transporter 3 (GLUT3), which allows for continuous import of glucose due to a high affinity (K_M_ below normal fasting blood glucose level) [6, 7]. Physiologic functions of nerves are fueled by ATP, which primarily is generated by glucose metabolism. Disruption of glucose metabolism underlies peripheral neuropathies, and diabetic hyperglycemia can cause up to fourfold increases in glucose levels. Persistent or repetitive excessive glucose uptake leads to altered intracellular glucose metabolism and neuronal damage often referred to as glucose neurotoxicity [8].

**Fig. 1:**
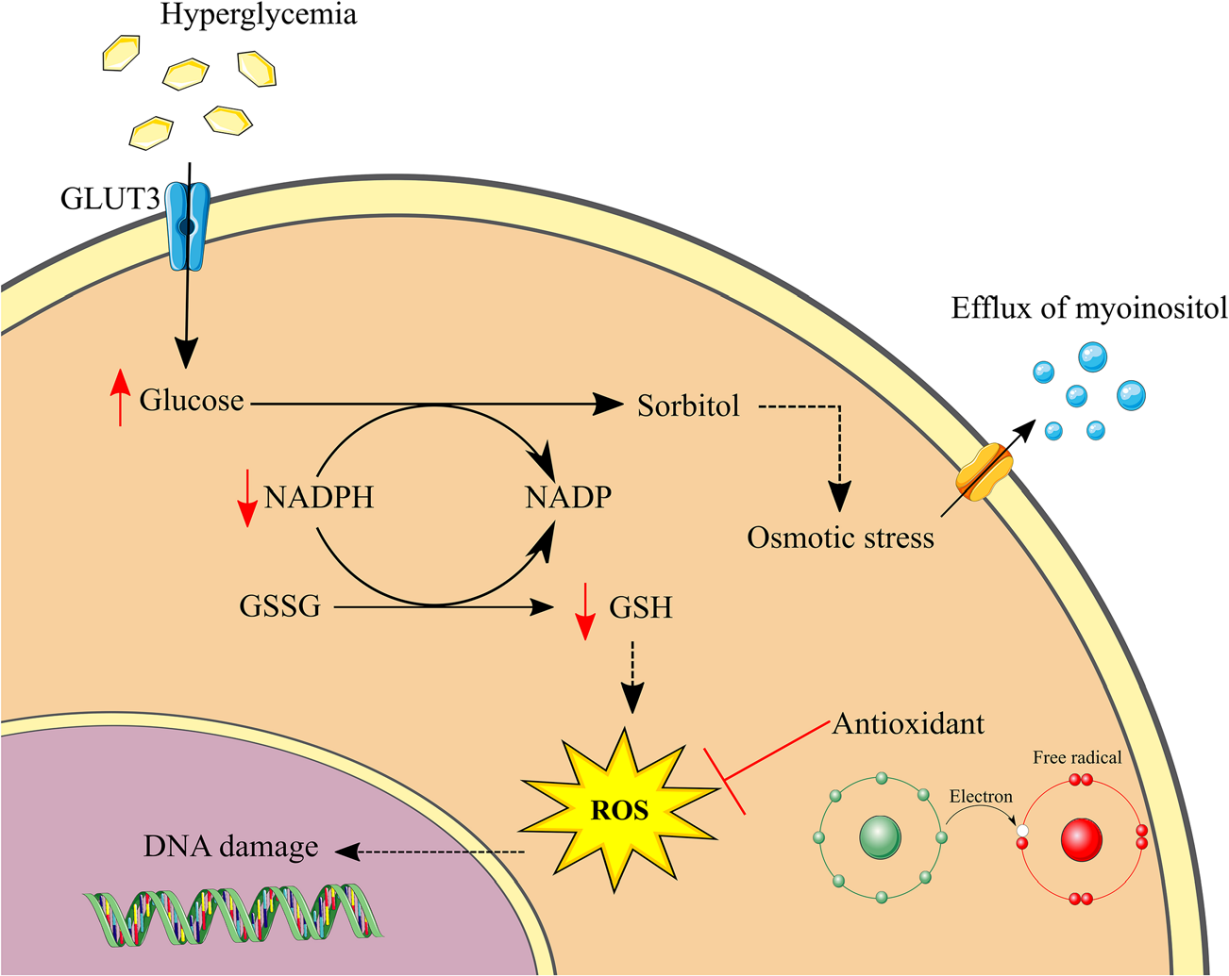
Overview of the polyol pathway. Hyperglycemia causes excessive glucose uptake in neurons through GLUT3. Consequently, increased levels of sorbitol lead to osmotic stress and ROS production due to decreased regeneration of the endogenous antioxidant GSH. GLUT3, glucose transporter 3; GSH, glutathione; GSSG, glutathione disulphide; NADP, nicotinamide adenine dinucleotide phosphate (oxidized); NADPH, nicotinamide adenine dinucleotide phosphate (reduced); ROS, reactive oxygen species

One metabolic consequence of excessive intracellular glucose is a shift towards the polyol pathway in which glucose is converted to sorbitol (Fig. 1). Increased sorbitol has two negative consequences on neuronal homeostasis. Firstly, it results in disturbances in the osmotic balance leading to compensatory efflux of myoinositol, which is essential for normal nerve function [9]. Secondly, the increased glucose-to-sorbitol conversion results in oxidative stress, which has been widely accepted as an important player in the pathogenesis of DSPN [10, 11]. Sorbitol production is facilitated by the enzyme aldose reductase and its cofactor NADPH. Under normal circumstances NADPH is also used in the regeneration of the endogenous antioxidant glutathione. The depleted cellular NADPH stores caused by excessive sorbitol production thus leads to impairments in the natural defense against oxidative stress with accumulation of regenerative oxygen species (ROS) within the cell as a consequence [9]. Additionally, hyperglycemia leads to increased production of pyruvate, which eventually causes the voltage gradient across the inner mitochondrial membrane to be increased, ultimately resulting in superoxide generation, oxidative stress, and mitochondrial injury [7]. This results in altered bioenergetics with reduced capacity of ATP production, which may cause cellular apoptosis and neuronal degeneration [11].

In addition, accumulation of glucose also induces the process of auto-oxidation in which glucose is converted to glyoxal. Glyoxal is one of three major advanced glycation end product (AGE) precursor molecules (the other two being methylglyoxal and 3-deoxyglucasone), which form spontaneous and irreversible bonds with proteins and lipids both intra- and extracellularly. These altered molecules, known as AGEs, interact with AGE receptors (RAGEs) thereby promoting activation of protein kinase C and transcription factor NF-κB leading to proinflammatory cytokine production. Furthermore, AGE–RAGE interaction promotes ROS production by depletion of glutathione and contributes to vascular dysfunction by reducing levels of nitric oxide necessary for vasodilation [12]. AGE formation thus contributes to endoneurial hypoxia and neuronal damage [13].

Development of DSPN is also influenced by altered lipid metabolism [14]. Oxidation of low-density lipoproteins produces substrates for the lectin-like oxLDL receptor (LOX-1), which upon activation initiates a signaling pathway resulting in increased ROS production [15].

Taken together, in recent years, DPN-focused research has evolved from a glucocentric viewpoint to a broader understanding of the underlying pathophysiology secondary to multiple linked metabolic and inflammatory insults including oxidative stress, reduced levels of nerve growth factors, and structural vascular changes, all of which contribute to a neurotoxic microenvironment [2, 9, 11].

## Prevention and Treatment of Diabetic Neuropathy

Despite efforts in early recognition and diagnosing of DSPN in order to slow the progression, currently, no effective treatment is available at a global level except for tight control of blood glucose [16]. In type 1 diabetes, appropriate blood glucose control aiming at near-normal levels reduces the incidence of DSPN substantially [5]. In type 2 diabetes, however, development of DSPN seems to be less influenced of blood glucose levels with only a relative risk reduction of less than 10% in well-regulated individuals [3, 17]. Lifestyle interventions with focus on increased exercise and healthy diet may have a beneficial effect in delaying nerve damage in diabetic subjects or even facilitating nerve fiber regeneration [18, 19].

Along with the increasing knowledge regarding pathogenesis of DSPN, experimental treatment options targeting the underlying molecular pathways have been investigated including attenuation of oxidative stress. The balance between generation and elimination of free radicals is crucial. Primary increase in radical generation, or a decrease in radical elimination from the cell, leads to oxidative cellular stress [20]. Experimental and clinical evidence support that the generation of reactive oxygen species (ROS) increases in both types of diabetes and that the development of DSPN is closely associated with oxidative stress [21, 22]. Thus, substances with antioxidative properties have gained interest in the attempt to slow progression or ultimately reverse development of DSPN. Additionally, compounds with neurosupportive characteristics may be utilized in the DSPN treatment regime [23].

Several naturally occurring bioactive compounds found in foods possess antioxidative and neuroprotective qualities, which may be utilized as a safe and effective treatment option for prevention of or even reversal of DSPN [24]. Thus, in the following, a short overview of dietary supplements suggested for treating DSPN is provided with focus on compounds studied in clinical trials during the last 5 years (Table 1).

**Table 1:** Overview of clinical studies in DPN during the last five years using dietary supplements targeting possible underlying molecular mechanisms as intervention

	Study design	Study population	Sample size	Intervention	Selected outcome(s)	Main significant findings	Limitations
Alpha-lipoic acid							
Agathos 2018 [27]	Single-arm Open label	Patients with either T1D or T2D and a DSPN diagnosis and one or more typical symptom of painful neuropathic pain	n = 72	600 mg/day of alpha-lipoic acid Duration: 40 days	NSS, SPNSQ, DN4 BPI, NPSI, SDS, PGI-I	Significant reductions self-reported neuropathy symptoms (NSS, SPNSQ and DN4) Significant improvement in quality of life (BPI (both pain severity and pain interference), total NPSI, SDS)	Not placebo-controlled No blinding No objective measures of DSPN Short intervention periods
Mrakic-Sposta 2017 [28]	Single-arm Open label	Patients with T2D and DSPN diagnosis	n = 12	1.6 mg/day of R (+)-thioic acid Duration: 120 days	Visual analog scale (subjective mood, general wellness and pain) NCT Antioxidant capacity and ROS detection in blood and plasma	Improved general wellness sensation Decreased pain sensation in upper limbs and lower limbs Increased motor nerve conduction velocity in superficial peroneal nerves and ulnar nerves. Increased sensory nerve conduction velocity in sural and median nerves Increased antioxidant capacity and decreased ROS production rate	Not placebo-controlled No blinding Low sample size
Garcia-Alcala 2015 [25]	Initial: Open-label Enriched: Randomized Open-label	Initial: Patients with T2D and symptomatic DSPN + TSS > 7 Enriched: Patients with a TSS reduction ≥3 after initial phase	Initial: n = 45 Enriched: 16 in active group 17 in withdrawal group	Initial: 3×600 mg/day of alpha-lipoic acid Duration: 4 weeks Enriched: 600 mg/day of alpha-lipoic acid Duration: 16 weeks	TSS Monofilament test, VPT, and ankle reflex	Initial: Decreased TSS in the responding group Increased VPT (right and left hallux) Enriched: Decreased TSS Reduced use of analgesic rescue medication	No blinding Small sample size
Curcumin							
Asadi 2019 [32]	RCT Double-blind	Patients with non-insulin-dependent T2D and DSPN diagnosis	n = 80 (40 in active group 40 in control group)	80 mg/day of Nano curcumin Duration: 8 weeks	TCNS including symptoms scores, sensory test scores, and reflex scores	Reduced total score of neuropathy, total reflex score and temperature sensitivity	Short intervention period
Vitamin E							
Ng 2020 [41]	RCT Double-blind	Patients with T2D	n = 80 (39 in active group, 41 in control group)	2×200 mg/day of Tocovid Duration: 8 weeks	NCT Serum NGF concentration	Increased sensory and motor conduction velocity of median, sural, and tibial nerves Increased levels of NGF in the active group	Short intervention period No subjective outcomes
Hor 2018 [42]	RCT Double-blind	Patients with diabetes and symptomatic DSPN	n = 300 (150 in each group)	2×200 mg/day of mixed tocotrienols Duration: 12 months	TSS, NIS NCT	No significant differences between groups after intervention	Multiple comorbidities and long-term disease duration may hamper reversibility of DSPN
Omega-3 fatty acid							
Lewis 2017 [46]	Single-arm Open-label	Patients with T1D and a Toronta Clinical Neuropathy Score ≥1	n = 40 (23 with signs of DSPN and 17 without)	2×5 mL/day of seal oil ω-3 poly-unsaturated fatty acids Duration: 12 months	NCT Temperature detection threshold Vibration perception threshold Reflex-mediated neurogenic vasodilation Heart rate variability Corneal nerve fiber length and density	Increased corneal nerve fiber length	Not placebo-controlled No blinding
Folic acid							
Mottaghi 2019 [51]	RCT Double-blind	Patients with diabetes and DSPN	n = 80 (40 in active group, 40 in control group)	1 mg/day of folic acid Duration: 16 weeks	NCT	Increased sensory sural amplitude Increased motor peroneal and tibial amplitude and velocity Decreased onset latency of motor peroneal and tibial nerves	No subjective outcomes
Vitamin D							
Ghadiri-Anari 2019 [58]	Single-arm Open label	Patients with T2D and pain DSPN	n = 60	50,000 IU/week of vitamin D_3_ Duration: 12 weeks	Evaluation of neuropathy by MNSI (questionnaires and physical examination)	Reduced MNSI questionnaire score and MNSI physical examination score	Not placebo-controlled No blinding
Alam 2017 [57]	Single-arm Open label	Patients with painful diabetic neuropathy	n = 143	Single intramuscular dose of 600,000 IU of vitamin D_3_ Duration: 20 weeks	NeuroQoL	Increased NeuroQoL subscale score of emotionnnal distress	Not placebo-controlled No blinding No objective measures of DSPN
Vitamin B12							
Didangelos 2021 [71]	RCT Double-blind	Patients with T2D and DSPN	n = 90 (44 in active group, 46 in control group)	1,000 μg/day of methylcobalamin Duration: 12 months	NCT VPT MNSI (questionnaires and physical examination)	Increased sensory sural nerve amplitude and velocity Improved VPT Improved MNSI-questionnaire, but not MNSI-examination	Single center study

BPI Brief Pain Inventory; DN4 Douleur neuropathique; MNSI Michigan Neuropathy Screening Instrument; NCT Nerve conduction testing; NeuroQoL Neuropathy specific quality of life; NGF Nerve growth factor; NIS Neuropathy Impairment Score; NPSI Neuropathic Pain Symptom Inventory; NSS Neuropathy Symptom Score; PGI-I Patient Global Impression of Improvement; RCT Randomized controlled trial; ROS Reactive oxygen species; SDS Sheehan Disability Scale; SPNSQ Subjective Peripheral Neuropathy Screen Questionnaire (SPNSQ); T1D Type 1 diabetes; T2D Type 2 diabetes; TCNS Toronto Clinical Neuropathy Score; TSS Total Symptom Score; VPT Vibration perception threshold

## Selected Dietary Supplements in Diabetic Neuropathy

### Alpha-lipoic Acid

The naturally occurring alpha-lipoic acid (ALA), also known as thioctic acid, serves as an important cofactor for enzymes required for generation of energy within the mitochondria. ALA also serves as a vital antioxidant capable of neutralization of ROS and scavenging of free radicals both intra- and extracellularly [25]. Additionally, ALA is capable of metal chelation, regeneration of endogenous antioxidants, and repair of oxidative damage [25, 26]. Only limited amounts of ALA are synthesized in the body, and supplementation through the diet is therefore necessary in order to reach sufficient levels [25].

ALA has been shown to increase glucose uptake and thereby improve glycemic control in diabetes [27, 28]. As such, ALA may have an indirect protective effect on the pathogenesis of DSPN, which is known to be influenced by long-term hyperglycemia. However, due to the potent antioxidative capacities, ALA has also been studied as a potential DSPN treatment option directly targeting the underlying pathophysiology [29]. A recent retrospective observational study showed a significant decrease in the self-reported and validated Neuropathy Symptom Score (NSS) after a minimum of 2 months ALA supplementation [30]. An improved NSS was also reported by a prospective single-arm intervention study after 40 days of ALA treatment. Furthermore, ALA supplementation reduced neuropathic pain and increased quality of life of included patients [31]. Improvements in objective measures of peripheral nerve function such as nerve conduction velocity have also been reported in a single-arm study [32]. The results of this study are, however, questionable due to the fact that no control-group and randomization were included. In a large randomized controlled trial with the active group receiving daily ALA supplementation (600 mg) for 4 years, a clinically meaningful improvement in DSPN was shown, but no change in nerve conduction testing was observed [33]. Interestingly, the beneficial effects on both antioxidant capacity and ROS production rate have been reported to be limited to short-term administration of ALA with a return to near-baseline levels after daily supplementation for 60 days [32]. This underlines the importance of further research including the optimal dose and duration of intervention.

### Curcumin

The spice turmeric has been used in traditional Asian medicine for thousands of years [34]. The active component, known as curcumin, can be isolated from the *C. longa* plant by drying and powdering of the roots [35]. Administration of curcumin as a nutritional supplement is complicated due to its hydrophobic nature resulting in low bioavailability. Consequently, synthetic formulations optimized regarding absorption rate and metabolism have been developed in order to overcome this issue [35, 36].

Several possible features of curcumin are of interest in regard to diabetic neuropathy. Anti-inflammatory, antioxidant, and neuroprotective capacities have been shown in animal studies [37–39]. A curcumin derivate, J147, has been shown to prevent oxidative stress through upregulation of adenosine 5′-monophosphateactivated protein kinase (AMPK) and associated decreased transient receptor potential A1 (TRPA1) expression [38]. Excessive activation of TRPA1 is present during oxidative stress and may induce hyperalgesia and neuroinflammation [40]. These findings are particularly interesting, since AMPK is known to play a vital role in the link between energy balance and metabolic response, and furthermore found to be dysregulated in diabetes [41]. Additionally, a blood glucose stabilizing effect of curcumin has been reported in both rodents and humans [34].

Data from clinical trials are sparse. A single randomized controlled trial has been conducted during the last 5 years. Here, 8-week supplementation of 80 mg nanocurcumin was shown to be effective in reducing DPSN measured by the validated Toronto Clinical Neuropathy Score [36]. This result is promising and should be validated in other randomized control trials.

### Vitamin E

The natural sources of vitamin E include green leafy plants, fruits, seeds, and plant oils. After its discovery in 1922, vitamin E has been studied in the context of several pathologies due to a well-documented and potent antioxidative capacity. Vitamin E can be divided into two groups known as tocopherols and tocotrienols each sub-divided in four isotypes. Both tocopherols and tocotrienols contain a hydroxyl group, capable of neutralization of ROS through hydrogen donation. Historically, tocopherols have been the most studied form of vitamin E, but recently, tocotrienols have gained increasing interest and are speculated to be superior in regard to antioxidative capacity [42].

Rodent models of diabetic neuropathy have shown increased antioxidant capacity as well as decreased proinflammatory cytokine levels in plasma after tocotrienol supplementation [43]. Recently, clinical studies have investigated vitamin E supplementation as DSPN treatment. In an open-label study, 92 patients diagnosed with DSPN were randomized to receive no intervention or vitamin E as an add-on to regular medications for 12 weeks. Subjects in the active group had a significantly lower total neuropathic pain score in addition to increases in self-reported physical health. When subdivided according to age, however, the improvement in neuropathic pain was restricted to the group above 50 years [44]. The open-label nature of this study is, however, a major risk factor for introducing bias in the subjective evaluation of treatment effect, and the results should therefore be interpreted with caution. Objective measures of neuronal function have been investigated in a randomized controlled trial in type 2 diabetes. Here, improvements in both sensory and motor nerve conduction velocity, but not amplitudes, after 8 weeks of vitamin E supplementation were reported. Furthermore, a significant higher serum concentration of NGF was observed in the active group after intervention [45].

Another randomized controlled trial on 300 diabetic subjects, however, failed to show any changes in both subjective and objective measures of DSPN after 12 months of vitamin E supplementation [46]. These contradictory results from recent clinical trials underline the need for further investigations on vitamin E supplementation in DSPN including dose and choice of isotype.

### Polyunsaturated Fatty Acids

Diabetes is associated with dysfunctional fatty acid metabolism resulting in impaired production rate of long-chain polyunsaturated fatty acids (PUFAs). Consequently, the composition of phospholipids in cellular membranes is disturbed [47]. Additionally, dyslipidemia characterized by increased serum levels of free fatty acids and triglycerides is common in type 2 diabetes, and an association to DSPN development has been observed [48]. Dietary interventions aiming at switching from high intake of saturated fatty acids to PUFAs or monounsaturated fatty acids (MUFA) may—at least theoretically—be beneficial in preventing neuronal damage. Certainly, in vitro studies have suggested the underlying mechanisms to be protective in mitochondrial function in both neurons and supportive Schwann cells [48, 49]. Another important mechanism involves oxygenated metabolites of PUFA-rich fish oils known as resolvins, which have shown to possess neuroprotective properties [50]. In the same line, animal studies with supplementation of fish oil have shown promising result regarding neuroprotective actions and antinociceptive effects on neuropathic pain [47, 51, 52]. Furthermore, a neuroregenerative potential of PUFAs has been shown in rodent models of DSPN with enhanced nerve conduction velocities and increased corneal nerve fiber length [53, 54].

Finally, in a single-arm clinical trial, 12 months of supplementation with omega-3 PUFAs increased corneal nerve fiber length. This effect was observed in patients both with and without signs of DSPN at baseline. However, no changes were shown in nerve conduction velocities, amplitudes, or sensory function [55]. These results should, however, be interpreted with caution due to the open-label, single-arm study design and the associated limitations. Nonetheless, the neuroregenerative potential of PUFAs found in preclinical studies is intriguing and should be investigated further in clinical randomized controlled trials.

### Folic Acid

Vitamin B9, better known as folate, is required for synthesis of pyrimidines and purines and thus essential for DNA replication and mitosis [56]. Folate is crucial during early embryogenesis, and maternal deficiencies during the first weeks of gestation are associated with a substantial increased risk of neural tube defects [57]. Due to its potent effect on neuronal growth during development, folate is interesting in regard to neuronal pathologies and has indeed been shown to be able to induce repair in the adult nervous system [57]. Folate must be introduced via the diet or from nutritional supplementation (often in the form of the synthetic folic acid) because the human organism is incapable of de novo synthesis [58].

In a study on streptozotocin-induced diabetic rats, daily supplementation of folic acid protected against DSPN development. This was shown both histologically, electrophysiological, and functionally. Furthermore, the study showed significantly increased levels of NGF in the sciatic nerve in the folic-acid-treated group compared to the diabetes group [59]. In addition to neurotrophic effects, folate has been shown to possess antioxidant properties [59].

In humans, a link between low serum folate levels and incidence of DSPN in type 2 diabetes has been proposed by a recent meta-analysis. Sub-group analysis, however, revealed that this observation was only present in the Chinese population, and not in the Caucasian [56]. In a randomized controlled trial, 16 weeks of folic acid supplementation (1 mg/day) significantly improved several objective components of nerve conduction including sensory amplitude (sural nerve) as well as motor amplitude and velocity (peroneal and tibial nerves) [60]. These findings are of high clinical interest, but confirmation from other cross-sectional studies and randomized controlled trials is needed.

### Vitamin D

Vitamin D is a fat-soluble compound best known for its effect on bone metabolism. However, the vitamin D receptor is found in various places including the bone, intestine, kidney, and neuronal tissue, thus underlining the importance of this substance in many physiological pathways [61].

A meta-analysis from 2015 found a three-fold increased risk of DSPN in diabetic patients with vitamin D deficiency compared to diabetic patients with adequate levels [62]. This is particularly interesting since low levels of vitamin D is common in diabetes [63, 64]. A biological link between DSPN and vitamin D may involve the peripheral glial cells, which support neuronal homeostasis by secretion of various neurotrophic factors including nerve growth factor (NGF). Through binding to receptors on the glial cell, vitamin D is capable of regulating NGF synthesis, and animal studies have shown that vitamin D deficiency leads to reduced levels of NGF [65].

Recently, two single-arm clinical studies have investigated the effect of vitamin D supplementation in painful DSPN. In one study, a single intramuscular vitamin D dose of 600,000 IU was administered to a cohort of 143 diabetic patients. Subsequently, participants were followed for 20 weeks. Neuropathy-specific quality of life improved significantly 12 weeks after the injection [66]. In the other study, an oral formulation of vitamin D was administered once weekly with a dose of 50,000 IU. After 12 weeks of intervention, significant improvements in DSPN evaluated by the Michigan Neuropathy Screening Instrument were seen, which consists of both subjective (questionnaire) and objective (physical examination) measures [67].

The lack of placebo arms in these studies is unfortunately major drawbacks to the study design, and future randomized controlled trials investigating both subjective and objective endpoints of neuronal function are thus needed in order to further explore the role of vitamin D in DSPN prevention or even reversal.

### Vitamin B12

Cobalamin better known as vitamin B12 is involved in DNA and protein synthesis. The natural sources include dairy products and meat [68]. Deficiency of this vitamin is highly prevalent in people with diabetes possibly due to decreased absorption capacity in the gastrointestinal tract caused by long-term metformin usage [69]. Insufficient levels of vitamin B12 are associated with several neurological pathologies including delirium, dementia, and neuropathy. Surveillance of vitamin B12 levels in people with diabetes is therefore crucial [70]. The effect of normalization of vitamin B12 levels in type 2 diabetes was investigated in a recent randomized controlled trial. Twelve months supplementation successfully increased B12 levels to normal values and improved both subjective and objective measure of DSPN [71]. Other clinical studies have reported similar results [72], and vitamin B12 status should therefore be incorporated in the standard diabetes checkup.

## Conclusion

With the increasing prevalence of patients with long-term diabetes and concomitant complications, demands for effective treatment options grow. Consequently, patients turn to the internet for inspiration regarding alternative over-the-counter therapies. Dietary supplements including antioxidants and vitamins have been suggested as possible treatment options specifically targeting the underlying molecular pathogenetic pathways. Many of these supplements have been used for several years and thus have well-described safety profiles. However, the proposed efficacy of dietary supplements for DSPN is often based on individual experiences rather than data from high-quality clinical trials. More research in this area is therefore encouraged, but several aspects should be considered carefully when planning future study designs.

Firstly, most dietary supplements are not subjected to the same rigorous regulations and controls (e.g., Good Manufacturing Practice) as the medical market, and quality and homogeneity of the investigated supplement should therefore be taken into consideration. Regarding DSPN, the conduction of clinical studies is complicated by several characteristics of the disease. Firstly, the disease progression is subtle and may extend over years or even decades eventually reaching a point-of-no-return in which the neuronal damages seem irreversible [9]. Inclusion of participants with longstanding diabetes and established DSPN may thus be incompatible with a positive outcome despite the use of an appropriate intervention. However, even early intervention may fail to prove effectiveness, mainly because of the slow progression of the disease in combination with the usually short-term intervention periods of clinical trials. Much thought should therefore be put into the optimal study design before conduction of any clinical trial investigating experimental therapies for DSPN. Another important aspect to keep in mind is the differences between type 1 and type 2 diabetes. Many studies include participants with a mixed distribution of diabetes type, and while both conditions may lead to hyperglycemia and associated neurotoxicity, the pathogenetic pathways vary substantially and may thus respond differently to the same intervention.

Subjective endpoints, e.g., patient-reported outcomes from questionnaire are vulnerable to recall bias and placebo response, especially in open-label studies. However, these patient-reported outcomes are significant for the patient and can reflect improvements in quality of life, which may vary from changes in objective outcomes of peripheral nerve function. In randomized controlled trials with double blinding, subjective outcomes can be attributed a high degree of validity and importance and should therefore be included. Regarding objective endpoints, nerve conduction velocities and action potentials are validated and robust measures of peripheral nerve function. These do, however, only reflect large fiber function, while measures of small fibers (e.g., monofilament testing and corneal confocal microscopy) may be more appropriate for detection of the early and regenerative potential of the intervention. Some studies have shown improvements in objective outcomes after supplementation with various compounds [32, 45, 55, 60], and further investigation and validation in larger randomized controlled trials are warranted.

In conclusion, the role of vitamins and supplements as possible therapy options for DSPN prevention or even reversal has shown promising results in several clinical studies. Further validation in high-quality randomized controlled trials is encouraged. Moreover, research regarding optimal initiation of intervention, duration, dose, and route of administration is warranted.
